# Detection of Cannabinoid Receptor Expression by Endometriotic Lesions in Women with Endometriosis as an Alternative to Opioid-Based Pain Medication

**DOI:** 10.1155/2022/4323259

**Published:** 2022-06-02

**Authors:** Sarah Allam, Elizabeth Paris, Itzel Lazcano, Pincas Bitterman, Sanjib Basu, James O'Donnell, Animesh Barua

**Affiliations:** ^1^Section of Emergency Medicine, Department of Medicine, 5656 South Maryland Avenue, Chicago, IL 60637, USA; ^2^Department of Anatomy and Cell Biology, Rush University Medical Center, Chicago, IL 60612, USA; ^3^Departments of Pathology, And Obstetrics & Gynecology, Rush University Medical Center, Chicago, IL 60612, USA; ^4^Department of Internal Medicine, Rush University Medical Center, Chicago, IL 60612, USA; ^5^Departments of Anatomy and Cell Biology, Pathology and Obstetrics & Gynecology, Rush University Medical Center, Chicago, IL 60612, USA

## Abstract

Emerging information suggests a potential role of medicinal cannabis in pain medication in addition to enhancing immune functions. Endometriosis is a disease of women of reproductive age associated with infertility and reproductive failure as well as chronic pain of varying degrees depending on the stage of the disease. Currently, opioids are being preferred over nonsteroidal anti-inflammatory drugs (NSAID) due to the latter's side effects. However, as the opioids are becoming a source of addiction, additional pain medication is urgently needed. Cannabis offers an alternative therapy for treating the pain associated with endometriosis. Information on the use and effectiveness of cannabis against endometriotic pain is lacking. Moreover, expression of receptors for endocannabinoids by the ovarian endometriotic lesions is not known. The goal of this study was to examine whether cannabinoid receptors 1 and 2 (CB1 and CB2) are expressed by ovarian endometriotic lesions. Archived normal ovarian tissues, ovaries with endometriotic lesions, and normal endometrial tissues were examined for the presence of endometrial stromal cells using CD10 (a marker of endometrial stromal cells). Expression of CB1 and CB2 were determined by immunohistochemistry, immunoblotting, and gene expression studies. Intense expression for CB1 and CB2 was detected in the epithelial cells in ovarian endometriotic lesions. Compared with stroma in ovaries with endometriotic lesions, the expression of CB1 and CB2 was significantly higher in the epithelial cells in endometriotic lesions in the ovary (*P* < 0.0001 and *P* < 0.05, respectively). Immunoblotting and gene expression assays showed similar patterns for CB1 and CB2 protein and *CNR1* (gene encoding CB1) and *CNR2* (gene encoding CB2) gene expression. These results suggest that ovarian endometriotic lesions express CB1 and CB2 receptors, and these lesions may respond to cannabinoids as pain medication. These results will form a foundation for a clinical study with larger cohorts.

## 1. Introduction

Endometriosis is a disorder of the reproductive system where endometrial tissue grows outside the uterus. It is associated with chronic pain, reproductive health, and infertility as well as social and psychological consequences [1]. It occurs in 6-10% of US women in the general population [2], and around 4 out of 100 women are hospitalized due to the condition each year [3]. Endometriosis is associated with pain of varying degree depending on the stage of the disease [4], and up to 71-78% of women with the disease may have chronic pelvic pain. Endometriosis affects the reproductive health and fertility of approximately 10-15% of women in their reproductive years [5], and of the total infertility cases in women, 20-50% is associated with endometriosis. The definitive diagnosis is based on symptoms in combination with imaging and/or biopsy [6]; however, most cases of endometriosis are misdiagnosed as the patients fail to report their symptoms correctly [1]. Thus, it may take up to 7 years from the time of incidence to the diagnosis of the disease [7]. Therefore, the burden on healthcare cost is enormous.

At this juncture, there is no curative method for endometriosis. Hormonal therapy, exploratory surgery, and pain medication are the currently available management options, all of which require high personal and public health cost [6, 8]. Surgical interventions may be an option for women at perimenopause or for women with unmanageable symptoms [6]. Thus, pain medication remains the principal mainstay for women of reproductive age. Nonsteroidal anti-inflammatory drugs (NSAID) are the common pain medication used for these patients [6]. The use of NSAIDs, however, appears to cause side effects particularly with long-term use which may lead to additional contraindications [9]. Narcotic prescription drugs including opioids are being used to avoid the side effects of NSAIDs for endometriosis. Unfortunately, availability and frequent use of opioids may result in addiction, which in turn encourages individuals to obtain them illegally [10]. Thus, additional pain medications are urgently required for the management of pain and chronic inflammation associated with endometriosis, raising the possibility that cannabinoids may be an alternative option to opioids.

Cannabinoids which bind with cannabinoid receptors are compounds found in the plant cannabis. There are two main cannabinoids: *Δ*9-tetrahydrocannabidiol (THC) and cannabidiol (CBD) [11]. CBD is recognized for its health benefits, including ameliorating inflammation, pain, anxiety, and seizures. Medical marijuana is typically grown to be high in CBD content and low in THC content so users can get the benefits without feeling the sense of euphoria. An earlier study suggested that targeting the endocannabinoid system was shown to be effective in alleviating neuropathic pain in animal models [11]. Furthermore, cancer-induced bone pain also shows signs of inflammatory and neuropathic pain [12]. Thus, endocannabinoids may target pain associated with inflammatory and neuropathic origins. Therefore, the endocannabinoid system may offer a potential option to replace the use of opioids in reducing the pain associated with the endometriosis. However, it is not well known if endometriotic lesions express receptors for endocannabinoids. Cannabinoid receptors are G protein-coupled cell surface receptors [13–15] and are of mainly two types including cannabinoid receptor 1 (CB1) and 2 (CB2) and are coded by *CNR1* and *CNR2* genes, respectively [16, 17]. Although the CB1 receptor is mainly expressed by the central nervous system, it may also be expressed in the lungs, kidneys, and liver. On the other hand, CB2 is expressed in various tissues [15] including the brain [15] and peripheral nervous system [18], members of the immune system [19, 20], and gastrointestinal system [20, 21]. In addition to its expression in peripheral and secondary lymphoid tissues including the spleen, tonsils, and thymus gland [20], CB2 receptors are also expressed by monocytes, macrophages, B cells, and T cells [19, 20].

Thus, information on the changes in expression of cannabinoid receptors in patients with ovarian endometriosis is very critical in mitigating inflammation as well as severe pain associated with this disease. The goal of this pilot study was to examine if ovarian endometriotic lesions express CB1 and CB2 cannabinoid receptors. The hypothesis of this study was that endometriotic lesions in the ovary express receptors for endocannabinoids. This hypothesis was tested by an exploratory design of experiments.

## 2. Methods and Materials

### 2.1. Clinical Specimens

Archived clinical specimens were collected from the Department of Pathology at Rush University Medical Center, Chicago, IL. Archived normal ovarian tissues (*n* = 15), ovaries with endometriotic lesions (*n* = 14), normal endometrial (*n* = 11), and normal myometrial (*n* = 6) tissues were collected from women who underwent surgery for ovarian endometriosis or prophylactic surgery for nonovarian or nonendometriotic conditions. All specimens were collected under the Institutional Review Board- (IRB-) approved protocol. Final diagnosis for the presence or absence of endometriosis were obtained from the Rush Department of Pathology.

### 2.2. Histopathological Examination of Clinical Specimens

The presence or absence of endometriotic lesions in selected tissues was determined by routine (H&E) staining using 5 *μ*m thick sections of paraffin-embedded tissue blocks.

### 2.3. Immunohistochemistry

Expression of CB1, CB2, and CD10 (an endometrial stromal cell marker) in paraffin sections from normal ovaries and endometrial tissues or ovarian tissues with endometriotic lesions was determined by immunohistochemistry as reported earlier [22]. Briefly, sections were deparaffinized with xylene and rehydrated using a descending series of ethanol followed by rinsing in DI (deionized) water. Antigens on each section were unmasked and retrieved by heating the sections in citrate solution (pH 6.0). Endogenous peroxidases in sections were neutralized by incubating with ice-cold 0.3% H_2_O_2_ in methanol for 15 min. Nonspecific binding of antibodies was blocked by incubating the sections with normal horse serum (Vector Laboratories, Burlingame, CA) for 30 min. Sections were then incubated overnight with primary antibodies, including rabbit anti-human cannabinoid receptor 1 (Millipore Sigma, St. Louis, MO), rabbit anti-human cannabinoid receptor 2 (Thermo Fisher Scientific, Waltham, MA), and mouse anti-human CD10 (Abcam, Cambridge, MA) at 1 : 100 dilutions. After incubation, sections were washed with phosphate buffered saline (PBS, 3 × 5 min) and incubated with anti-rabbit/mouse universal biotinylated secondary antibodies for 1 hour (Vector Laboratories, Burlingame, CA). After washing with PBS (3 × 5), sections were then incubated with peroxidases conjugated with avidin for 1 hour (Vector Laboratories, Burlingame, CA). Sections were then washed with PBS, and immunoreactions on the sections were visualized by incubating with 3,3′diaminobenzidine (DAB) containing H_2_O_2_ substrate under a light microscope. Once the reaction was complete, sections were washed, counterstained with hematoxylin, dehydrated with an ascending series of ethanol, placed in xylene, and mounted with an organic mounting media and covered with a cover slip and dried overnight in an oven. Sections were later examined under a light microscope attached to a computer-assisted software program for imaging (MicroSuiteTM version 5, Olympus American, Inc., Center Valley, PA). Images from 3-5 areas at 40x magnification in a section containing the approximately highest population of immunostained cells, or stronger staining intensities were taken and archived as reported earlier [23]. Double label immunostaining was performed to understand the morphology of immunopositive cells (stromal/immune cells and epithelial cells of endometriotic lesion or normal epithelium). Double label immunostaining was performed to determine the localization and type of cells expressing both CB1 and CD10 and cells expressing both CB2 and CD10. Sections were first immunostained for either CB1 or CB2, and immunoreactions were visualized with DAB under a light microscope as mentioned above. Sections were washed in PBS for 15 min to remove unbound DAB substrates and incubated for 2 hours at room temperature with anti-CD10 antibody at a 1 : 100 dilution. Sections were processed as described for single immunostaining with the exception that DAB-containing nickel peroxide substrate was used for visualization. Sections were dehydrated and mounted as described above. Examination of double-labeled immunostained cells was performed, and 5 images at 40x magnification containing approximately the highest double-labeled cells were taken and archived as reported earlier [24].

### 2.4. Counting of Immunostaining/Frequency of Immunopositive Cells

Archived images were examined, and the intensities of CB1 and CB2 immunostaining and the frequency of CB2 label immunostained cells were determined using a computer-assisted software program (MicroSuiteTM version 5, Olympus American Inc., Center Valley, PA). The mean intensity of CB1 and CB2 immunostaining or frequency of immunopositive CB2 cells in a section was determined as the arbitrary values from the 3-5 areas at 40x magnification. Intensity values were converted to express as intensities in 20,000 *μ*m^2^ area of tissue. Intensities and/or frequencies of CB1 and/or CB2 expressing cells among different groups were determined and reported. As the double label immunostaining was performed to understand the morphology of cells expressing CB1 or CB2, frequency of these cells was not quantified.

### 2.5. Western Blotting

Immunohistochemical expression of CB1 or CB2 was confirmed by immunoblotting of homogenates collected from representative specimens of normal ovaries, ovaries with endometriotic lesions, and normal endometrial or myometrial tissues as reported earlier [14]. Briefly, proteins from each selected specimen were resolved and separated in 10% gel (Bio-Rad, Hercules, CA). Proteins in gel were then transferred to nitrocellulose membrane (Bio-Rad, Hercules, CA). Membranes containing proteins were then blocked with 1% BSA solution followed by incubating overnight with primary antibodies (mentioned above) at a 1 : 1000 dilution. Membranes were then incubated with anti-rabbit secondary antibodies conjugated with horseradish peroxidase for one hour. Immunoreactions on the membranes were detected as chemiluminescent products using Immobilon Forte Western HRP substrate (Millipore-Sigma, St. Louis, MO), and images were captured by ChemiDoc XRS (Bio-Rad Laboratories, Hercules, CA). Images were archived for analysis later. Quantification of Western blot signals for CB1 and CB2 was performed from the images using the analysis® getIT! Software (Olympus Soft Imaging Solutions Corporation, Lakewood, CO) as reported earlier [25]. Intensity of signals of CB1 or CB2 protein expression in immunoblotting was determined. Signal intensities are presented as arbitrary values (mean ± SEM) in 20,000 *μ*m^2^ area as reported earlier [25].

### 2.6. Gene Expression Assays

Changes in the expression of *CNR1* and *CNR2* genes (genes encoding the CB1 and CB2 proteins) by normal tissues (including normal ovaries, normal endometrium, and myometrium) and tissues with endometriotic lesions were assessed by reverse-transcriptase polymerase chain reaction (RT-PCR) and quantitative RT-PCR (qRT-PCR) as reported earlier [26]. *β*-Actin was used as control. The following primers were used (5′ ➔ 3′):

Cannabinoid receptor 1: *F*: CTGGAACTGCGAGAAACTGC, *R*: AGAAGCAGTACGCTGGTGAC

Cannabinoid receptor 2: *F*: ACTCCATGGTCAACCCTGTC, *R*: GATCTCGGGGCTTCTTCTTT

*β*-Actin: *F*: CCACCATGTACCCTGGCATT, *R*: GTACTTGCGCTCAGGAGGAG

### 2.7. Statistical Analysis

Significant differences in the intensities of CB1 and CB2 expression between the endometriotic lesions and stromal tissues surrounding the lesion were determined by paired *t*-test, and comparisons of Western blot signals across all tissue groups were performed using one-way ANOVA using GraphPad Prism version 6.0 (GraphPad Software Inc., La Jolla, CA). Differences were considered significant when *P* < 0.05. Similarly, significant differences in the frequency of CB2-expressing cells and differences in fold changes in *CNR1* and *CNR2* gene expression were determined, and significance was taken when *P* < 0.05.

## 3. Results

### 3.1. Microscopic Features

**Figure 1:**
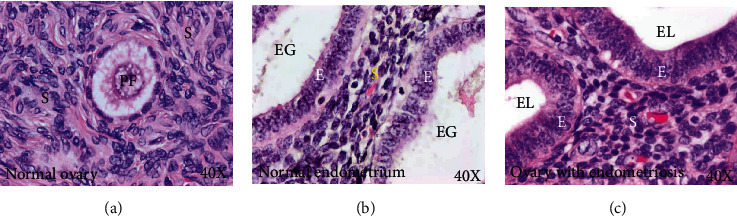
Microscopic presentation of normal ovary and ovary with endometriosis. (a) Section of a normal ovary showing a primordial follicle (PF) embedded in the ovarian stroma (S). (b) Section of normal endometrium showing closely packed endometrial glands (EG) containing a single layer of epithelium (E) arranged in the stroma. (c) Section of an ovary with endometriosis showing lesions with closely packed glands displaying a single layer of epithelium similar to endometrial glands present in normal endometrium. E = epithelium; EG = endometrial glands; EL = endometriotic lesion; PF = primordial follicle; S = stroma; 40x = magnification.

Routine staining with hematoxylin and eosin showed similarities in microscopic features between the ovarian endometriotic lesions and normal endometrium. As observed in normal endometrium, glands lined by a single layer of well demarcated epithelium surrounding the glandular lumina were seen in the stroma of the ovary containing the endometriotic lesions (Figure 1).

### 3.2. Expression of CD10

**Figure 2:**
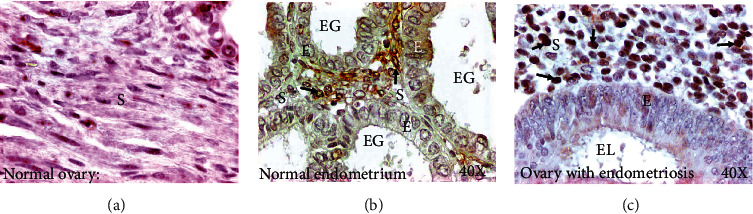
Expression of CD10 by the endometrial stromal cells in normal ovaries and ovary with endometriosis. (a) Section of a normal ovary. No CD10-positive stromal cells are seen. (b) Section of an endometrium showing stromal cells expressing CD10. However, epithelial cells in the endometrial glands were negative for CD10 expression. (c) Section of an ovary with endometriosis showing intense expression for CD10 by many stromal cells. Similar to normal endometrium, epithelial cells in endometriotic lesions (EL) did not show CD10 expression. E = epithelium; EG = endometrial glands; EL = endometriotic lesion; S = stroma; 40x = magnification. Arrows indicate examples of immunopositive CD10 cells.

CD10 is considered as a reliable immunohistochemical marker of endometrial stroma. Stromal cells in ovaries with endometriotic lesions showed strong immune reactivity for CD10 (Figure 2). Diffuse patterns of staining by the stromal cells were also seen occasionally in ovaries with endometriotic lesions. In contrast, no staining for CD10 was present in the epithelial cells of the endometriotic lesions (Figure 2). These results confirmed endometriosis in the ovary.

### 3.3. Expression of CB1 and CB2 Receptors

#### 3.3.1. Immunohistochemical Staining of CB1

**Figure 3:**
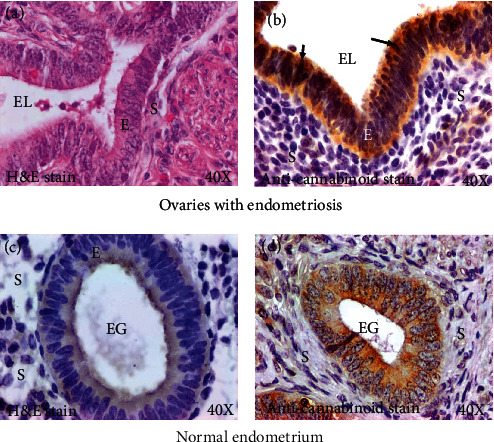
Expression of cannabinoid receptor 1 (CB1) by the epithelial cells or stromal cells in ovaries with endometriosis. (a) Section of an ovary with endometriosis stained with hematoxylin and eosin showing lesions with glands. (b) Section of an ovary with endometriosis showing intense expression for CB1 by epithelial cells of endometriotic lesions (EL). (c) Section of a normal endometrium showing an endometrial gland (EG) containing a single layer of epithelium. (d) Section of a normal endometrium showing moderate expression for CB1. E = epithelium; EG = endometrial glands; EL = endometriotic lesion; S = stroma; 40x = magnification. Arrows indicate examples of immunopositive CB1 cells.

Epithelial cells of the endometriotic glands in ovaries with endometriosis showed intense staining for CB1 (Figure 3). No staining was observed for CB1 in the stroma or ovarian surface epithelial cells in normal ovaries. Compared with the epithelial cells in the endometriotic lesions in ovaries with endometriosis, endometrial glands in normal endometrium showed relatively weaker staining for CB1 (Figure 3). Stromal cells in the ovaries with endometriotic lesions and in normal endometrium showed occasional diffusive patterns of staining for CB1.

**Figure 4:**
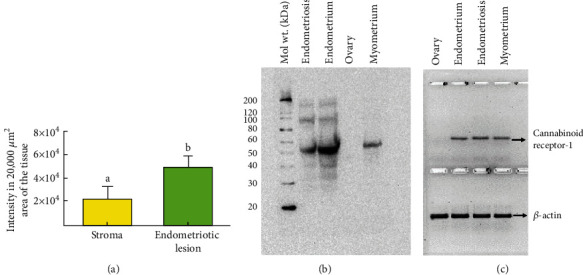
Expression of cannabinoid receptor 1 (CB1) in ovaries with endometriosis. *Top panel*: (a) intensity of expression of CB1 by endometriotic lesions in ovaries with endometriosis. Compared with the stroma, the intensity of CB1 expression is significantly higher in the epithelial cells in endometriotic lesions. Bars with different letters denote significant difference between them (*P* < 0.0001). *Bottom panel*: (b) CB1 protein expression: immunoblotting detected CB1 protein of approximately 50 kDa in tissue extracts from ovaries with endometriosis, endometrial, and myometrial extracts. In contrast, extracts from normal ovaries showed no expression for CB1. (c) Gene expression studies detected expression of *CNR1* gene in all tissues examined except in normal ovarian tissues.

Intensity of CB1 expression in the stroma of ovaries with endometriotic lesions was 2.11 × 10^4^ ± 1.09 × 10^4^ in 20,000 *μ*m^2^ area of the tissue. In contrast, the intensity of CB1 expression increased significantly (4.85 × 10^4^ + 0.9 × 10^4^ in 20,000 *μ*m^2^ area of the tissue) (*P* < 0.0001) in the endometriotic glands in ovaries with endometriosis (Figure 4, top panel).

#### 3.3.2. Immunoblotting for CB1

Overall, immunoblotting showed an immunoreactive band of approximately 50 kDa for CB1 (Figure 4(b), bottom panel). As expected, no band for CB1 was detected in normal ovarian extracts, whereas extracts from normal endometrium, myometrium, and ovaries with endometriotic lesions showed immunoreactive bands for CB1 of various intensities. Normal endometrial tissues showed stronger expression of CB1 (2.12 × 10^5^ ± 0.08 × 10^5^ in 20,000 *μ*m^2^ of the blot, *P* < 0.0001) followed by ovaries with endometriosis (1.23 × 10^5^ ± 0.14 × 10^5^ in 20,000 *μ*m^2^ of the blot, *P* < 0.001) and myometrium (0.75 × 10^5^ ± 0.02 × 10^5^ in 20,000 *μ*m^2^ of the blot, *P* < 0.05) (Supplementary figure 1).

#### 3.3.3. Gene Expression Studies for CNR1

Strong expression of *CNR1* gene was observed in normal endometrium (5.15-fold, *P* < 0.001) followed by ovaries with endometriosis (4.06-fold, *P* < 0.01) and myometrium (2.38-fold, *P* < 0.05). In contrast, expression of *CNR1* gene was not detected in specimens from normal ovaries (Figure 4(c), bottom panel) (Supplementary figure 2).

Compared with normal ovaries, changes in patterns of *CNR1* gene expression in ovaries with endometriotic lesions, as determined by semi- and quantitative RT-PCR, support the immunohistochemical and immunoblotting observations mentioned above.

#### 3.3.4. Immunohistochemical Staining of CB2

**Figure 5:**
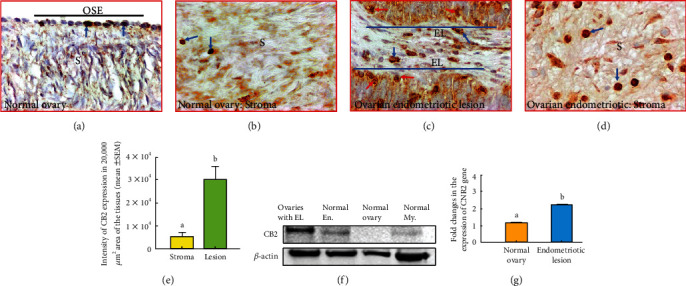
Expression of cannabinoid receptor 2 (CB2) by the epithelial cells or stromal cells in ovaries with or without endometriosis. (a) Section of a normal ovary showing occasional staining by a few epithelial cells of the ovarian surface. (b) Section of a normal ovary showing CB2 staining by a few immune cell-like cells in the stroma (S). (c) Section of an ovary with endometriosis showing CB2 expression by the cell membrane and nuclei of the epithelial cells in endometriotic lesions (EL). Interlesion stromal cells also showed staining for CB2. (d) Section of an ovary with endometriosis showing CB2 expression by many immune cell-like cells in the stroma. (e) Intensity of the CB2 expression was significantly higher in the epithelial layers in endometriotic lesions than the stroma in ovaries with endometriosis (*P* < 0.05). (f) CB2 protein expression: immunoblotting detected CB2 protein of approximately 40 kDa in tissue extracts from ovaries with endometriosis, normal endometrial, and myometrial tissue extracts. Extracts from normal ovaries showed no or very weak expression for CB2. In contrast, ovaries with endometriosis showed strong signal for CB2. (g) Gene expression studies detected strong expression for *CNR2* gene in ovaries with endometriosis, while it was almost undetectable in normal ovarian tissues. EL = endometriotic lesions; OSE = ovarian surface epithelial cells; Normal En = normal endometrium; Normal My = normal myometrium; S = stroma; 40x = magnification. Arrows indicate examples of immunopositive CB2 cells in the normal OSE cells and immune cell-like stromal cells (blue arrows) and nuclei (red arrows) of the endometriotic cells. Bars with different letters denote significant difference between them.

Occasional staining for CB2 was observed in the epithelial cells of the ovarian surface in normal ovaries (Figure 5(a)). In contrast, strong expression for CB2 was detected in the epithelial cells of the endometriotic lesions in ovaries with endometriosis (Figure 5(c)). In addition, nuclei of the epithelial cells of the endometriotic lesions also showed strong staining for CB2 expression (Figure 5(c)). Furthermore, immune cell-like cells in the stroma showed expression for CB2 in ovaries with and without endometriosis (Figures 5(b) and 5(d)).

The intensity of CB2 expression in the stroma of ovaries containing endometriotic lesions was 0.54 × 10^4^ ± 0.10 × 10^4^ in 20,000 *μ*m^2^ area of the tissue. The intensity of CB2 expression increased significantly to 3.04 × 10^4^ ± 0.66 × 10^4^ in 20,000 *μ*m^2^ area of the tissue in the epithelial lining of the endometriotic glands in ovaries with endometriosis (*P* < 0.05) (Figure 5(e)).

The mean frequency of CB2-expressing immune cell-like cells in the stroma of ovaries with endometriosis was approximately 4.5 ± 0.44 cells (mean ± SEM) in 20,000 *μ*m^2^ area. In contrast, the frequency of CB2-expressing immune cell-like cells in the endometriotic lesions of ovaries with endometriosis was 16.3 ± 2.11 cells (mean ± SEM) in 20,000 *μ*m^2^ area (*P* < 0.01) (Supplementary Figure 3).

#### 3.3.5. Immunoblotting for CB2

Immunoblotting detected a band of approximately 40 kDa for CB2 (Figure 5(f)). Similar to CB1, very weak or no band for CB2 was detected in normal ovarian extracts, whereas extracts from normal endometrium, myometrium, and ovaries with endometriotic lesions showed immunoreactive bands for CB2 of various intensities. Ovarian endometriotic lesions showed relatively stronger expression of CB2 (1.0 × 10^5^ ± 0.11 × 10^5^ in 20,000 *μ*m^2^ of the blot, *P* < 0.0001) followed by normal endometrium (0.89 × 10^5^ ± 0.07 × 10^5^ in 20,000 *μ*m^2^ of the blot, *P* < 0.0.001) and myometrium (0.26 × 10^5^ ± 0.08 × 10^5^ in 20,000 *μ*m^2^ of the blot, *P* > 0.05) when compared to normal ovaries (Supplementary Figure 4).

#### 3.3.6. Gene Expression Studies for *CNR2*

Quantitative (Figure 5(g)) and semiquantitative (Supplementary figure 5) gene expression assays showed very weak *CNR2* gene expression in normal ovaries. Compared with normal ovary (1.00), expression of *CNR2* was significantly higher in endometriosis (1.94 − fold ± 0.05, *P* < 0.05) (Figure 5(g)).

## 4. Discussion

This study reports, for the first time, expression of cannabinoid receptors 1 and 2 (CB1 and 2) in ovaries with endometriosis. This study further showed that compared with stromal tissues surrounding the lesion, the intensities of CB1 and CB2 expression were significantly higher in endometriotic lesions in the ovary. As endocannabinoid receptors are associated with the mediation of endometriosis-associated chronic pain and immune response, these results may be helpful in designing endometriotic lesion-specific targeted pain medications as well as anti-inflammatory therapeutics.

Currently, no curative approach is available for endometriosis, and in most cases, it is mainly managed by 2 modalities: efforts to ameliorate the pain and treating the patients with infertility [27]. As menopause ablates the incidence or recurrence of the disease, induction of menopause by surgical removal of the ovaries is an option for women suffering from endometriosis-related infertility, or women at their perimenopause suffering from severe symptoms [28, 29]. In contrast, relieving the pain and/or limited surgery in an effort to halt the progression of the disease while allowing the ovaries to function is the only option for patients of reproductive age if fertility is desired [30, 31]. Thus, information on molecular pathways involved in effective mediation of pain killers as well as immunotherapeutics including the expression of CB1 and CB2 as observed in this study is critical.

This study showed strong staining for CD10 by the stromal cells in ovaries with endometriotic lesions. CD10 is an established immunohistochemical marker for endometrial stromal cells [32]. Thus, this result confirms that endometriotic lesions in the ovary are accompanied with stromal cells of endometrium origin. Endometriosis-associated pain is complex and has heterogeneous origins. The lesions are stimulated by cyclic hormonal changes during which blood may accumulate locally. Endometriotic lesions in the ovary become blood-filled sac-like cysts which contain endometrium-like tissue [33]. If the accumulated blood is not removed either by the circulatory or lymphatic system, it may lead to swelling and inflammation with the activation of proinflammatory cytokines, resulting in the development of pain [34]. Ovarian endometriosis is classified by the American Society of Reproductive Medicine (ASRM) [35] as a stage III disease when lesions in the ovaries are accompanied with adhesion of the ovaries to other tissues including the fallopian tubes, peritoneum, or urinary bladder. These tubo-ovarian complexes are a source of pain during the entire menstrual cycle [34].

Nonsteroidal anti-inflammatory drugs (NSAIDs) are popularly used as painkiller in combination with other approaches [36]. The use of NSAIDs, however, appears to present side effects and contraindications with long-term use [9]. In such cases, narcotic prescription drugs have been suggested as an alternative measure.

Opioids, a class of narcotic prescription drug used as analgesic, exert their action in a similar manner as naturally occurring pain-reducing endorphins. Much of the opioid use is associated with patients with chronic noncancer pain with the risk of potential addiction and dependence [10]. Opioid misuse has become an epidemic and is associated with morbidity and mortality due to overdose [10]. Cannabinoids offer an alternative to the opioid epidemic.

Cannabinoids are compounds found in cannabis. The endocannabinoid system (ECS) consists of endogenous ligands, receptors, and enzyme that are required for ligand biosynthesis and their degradation. Endocannabinoids are amides, esters, and ethers of long chain polyunsaturated fatty acids. Cannabinoid receptors 1 and 2 are two G protein-coupled cell surface receptors that bind with endogenous or exogenous endocannabinoids with similar affinity. The present study showed strong immunohistochemical expression of CB1 and CB2 by the glands of endometriosis in ovaries, whereas little to no expression was observed in ovarian tissues in healthy subjects. Enhanced immunohistochemical detection of CB1 and CB2 expression in ovarian endometriotic lesions was supported by increased mRNA (RT-PCR) and protein (Western blotting) expression in these tissues. Similar observations in endometrial cells of the uterus were reported by others [37, 38]. Thus, cannabinoids may offer an alternative to opioids for managing the pain associated with ovarian endometriosis. Moreover, cannabinoids have also been reported to be an antiproliferative agent making it an effective and desired analgesic with potential antigrowth properties for ovarian endometriotic patients [39].

CB2 receptors have been suggested to be associated with various cellular processes including apoptosis, cell migration, and immune function [19]. Depending on its binding sites, increased expression of CB2 modulates cellular functions in immune cells by regulating the levels of cAMP [19]. Inhibition of cAMP signaling through CB2 receptors (via inhibitory protein G*α_i_*) results in the reduction of immune regulatory genes [40] leading to immunosuppression [41, 42]. In contrast, increase in cAMP synthesis stimulated by CB2-agonists (through stimulatory protein G*α*_s_) has shown to induce IL-16 and IL-10 production [43]. Thus, CB2 agonists may also be useful for treatment of inflammation and pain and are currently being investigated, in particular, for forms of pain that do not respond well to conventional treatments, such as neuropathic pain [43, 44]. This study showed significant increase in expression of CB2 in ovaries with endometriotic lesion compared to normal ovaries, suggesting that CB2 may be a potential target for immunotherapies against ovarian endometriosis.

Smaller sample size is a limitation of this study. However, studies on ovaries with endometriosis are very limited as compared with endometriosis involving other tissues. Pain management and/or the immunotherapies may be considered as pragmatic option(s) for the treatment of endometriosis in women of reproductive age. The results of this study will be a foundation for a clinical study with a larger cohort to determine the feasibility of cannabinoids as effective painkillers and/or immune modulators for these patients.

## Data Availability

All data supporting the conclusions of this article are included in the article.

## Supplementary Materials

Supplementary Materials Supplementary figure 1: intensity of CB1 protein expression in immunoblotting from tissues with or without endometriosis. As expected, normal endometrium showed highest intensity of signal for CB1 expression followed by ovaries with endometriosis and normal myometrium. In contrast, normal ovaries showed very weak or no signal for CB1 in immunoblotting. Bars with different letters are significantly different (*P* < 0.05). Supplementary figure 2: changes in *CNR1* gene expression during the development of ovarian endometriosis. Expression of *CNR1* gene was least in normal ovaries. In contrast in qRT-PCR, *CNR1* expression was significantly higher in normal myometrium (more than 2 fold (*P* < 0.05) and 4-5-fold in normal endometrium as well as in ovaries with endometriosis (*P* < 0.001)). Supplementary figure 3: localization of CB2-expressing cells in ovaries with endometriosis. (A) Section of an ovary with endometriosis immunostained for CB2 expression. Immune cell-like cells are seen to express CB2 in the endometriotic lesion (EL) and in the stroma (arrows indicate the examples of immunopositive CB2 expressing immune cell-like cells). (B) The frequency of CB2-expressing immune cell-like cells in endometriotic lesions was significantly higher than that in stroma. EL = endometriotic lesions; G = gland; 40x = magnification. Arrows indicate examples of immunopositive CB2 cells. Supplementary figure 4: intensity of CB2 protein expression in immunoblotting from tissues with or without endometriosis. As expected, ovaries with endometriosis and normal endometrium showed strong intensity of signal for CB2 expression followed by normal myometrium. In contrast, normal ovaries showed very weak or no signal for CB2 in immunoblotting. Bars with different letters are significantly different (*P* < 0.05). Supplementary figure 5: changes in *CNR2* gene expression during the development of endometriosis. Expression of *CNR2* was very weak in normal ovary and myometrium. In contrast, expression of *CNR2* was stronger in normal endometrium and ovaries with endometriosis. *β*-Actin was used as housekeeping gene, and its expression was similar in all tissues.
