# Supplementary material for: Detection of Cannabinoid Receptor Expression by Endometriotic Lesions in Women with Endometriosis as an Alternative to Opioid-Based Pain Medication
Source: J Immunol Res. 2022 Jun 2;2022:4323259. doi: 10.1155/2022/4323259 (PMC9184153; doi:10.1155/2022/4323259)
Supplement: Supplementary Materials — Supplementary figure 1: intensity of CB1 protein expression in immunoblotting from tissues with or without endometriosis. As expected, normal endometrium showed highest intensity of signal for CB1 expression followed by ovaries with endometriosis and normal myometrium. In contrast, normal ovaries showed very weak or no signal for CB1 in immunoblotting. Bars with different letters are significantly different (P < 0.05). Supplementary figure 2: changes in CNR1 gene expression during the development of ovarian endometriosis. Expression of CNR1 gene was least in normal ovaries. In contrast in qRT-PCR, CNR1 expression was significantly higher in normal myometrium (more than 2 fold (P < 0.05) and 4-5-fold in normal endometrium as well as in ovaries with endometriosis (P < 0.001)). Supplementary figure 3: localization of CB2-expressing cells in ovaries with endometriosis. (A) Section of an ovary with endometriosis immunostained for CB2 expression. Immune cell-like cells are seen to express CB2 in the endometriotic lesion (EL) and in the stroma (arrows indicate the examples of immunopositive CB2 expressing immune cell-like cells). (B) The frequency of CB2-expressing immune cell-like cells in endometriotic lesions was significantly higher than that in stroma. EL = endometriotic lesions; G = gland; 40x = magnification. Arrows indicate examples of immunopositive CB2 cells. Supplementary figure 4: intensity of CB2 protein expression in immunoblotting from tissues with or without endometriosis. As expected, ovaries with endometriosis and normal endometrium showed strong intensity of signal for CB2 expression followed by normal myometrium. In contrast, normal ovaries showed very weak or no signal for CB2 in immunoblotting. Bars with different letters are significantly different (P < 0.05). Supplementary figure 5: changes in CNR2 gene expression during the development of endometriosis. Expression of CNR2 was very weak in normal ovary and myometrium. In contrast, expression of CNR2 [file 4323259.f1.pdf]

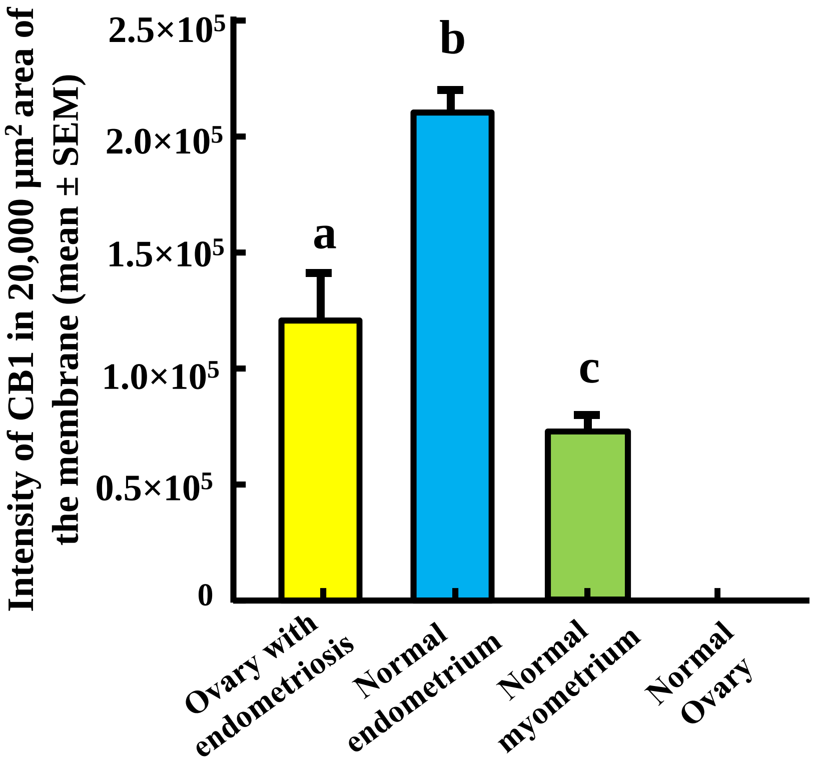

Supplementary figure 1.

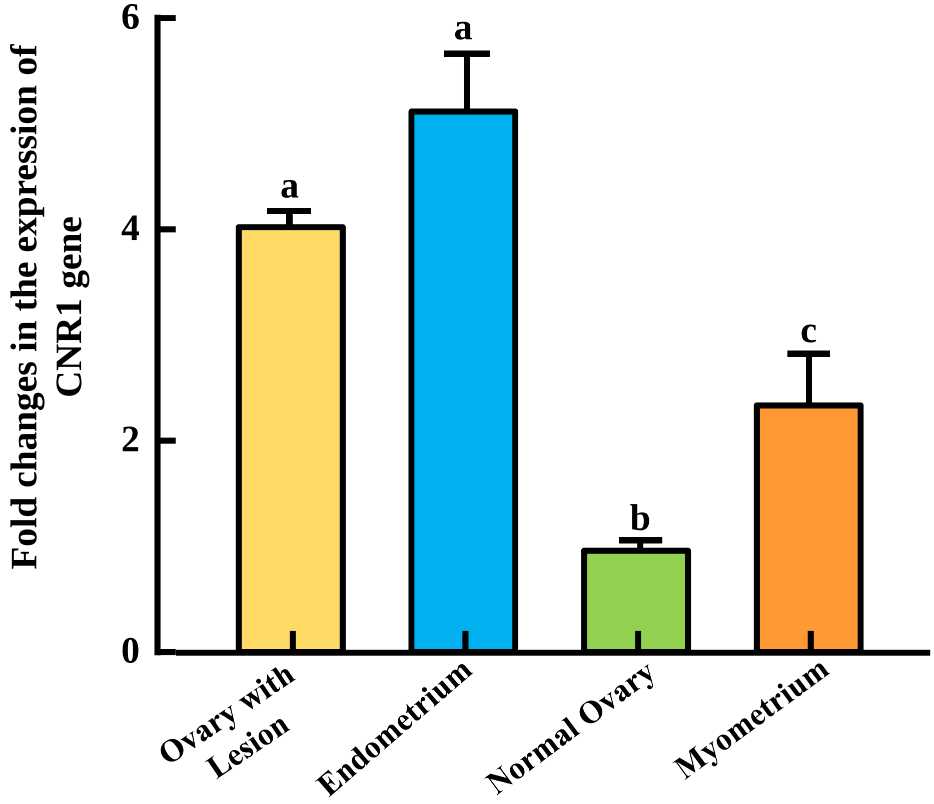

Supplementary figure 2

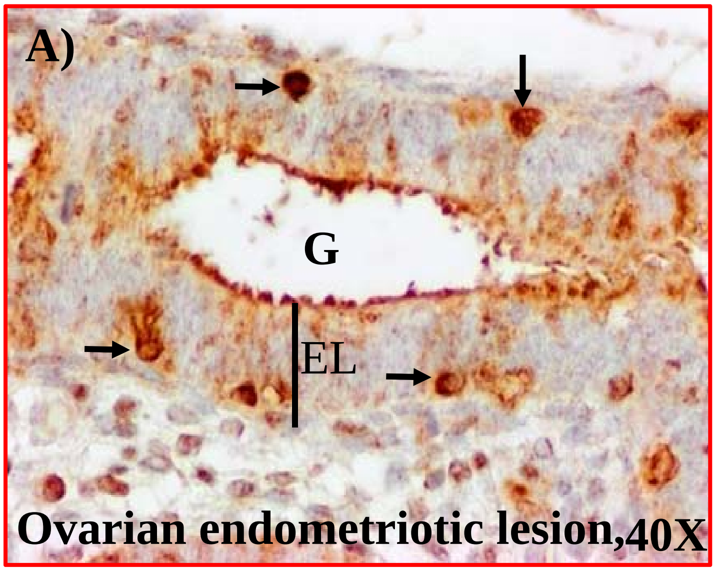

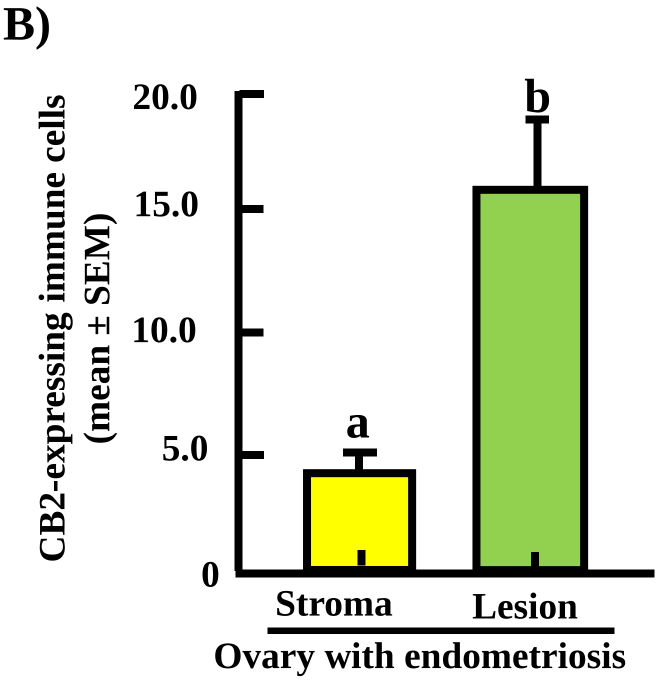

Supplementary figure 3

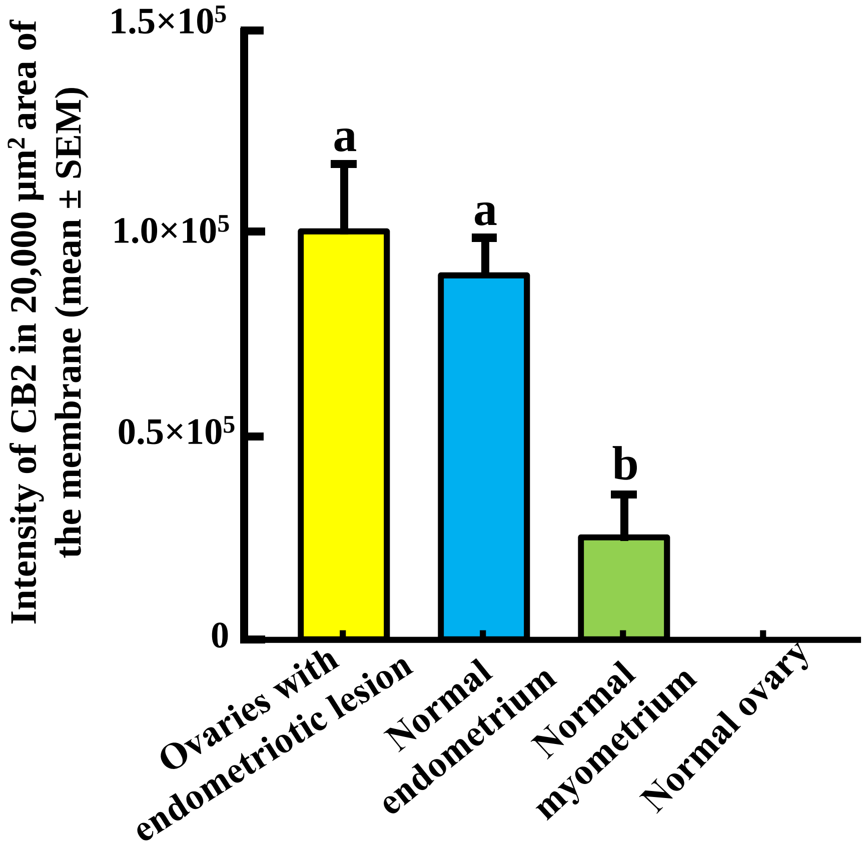

Supplementary figure 4

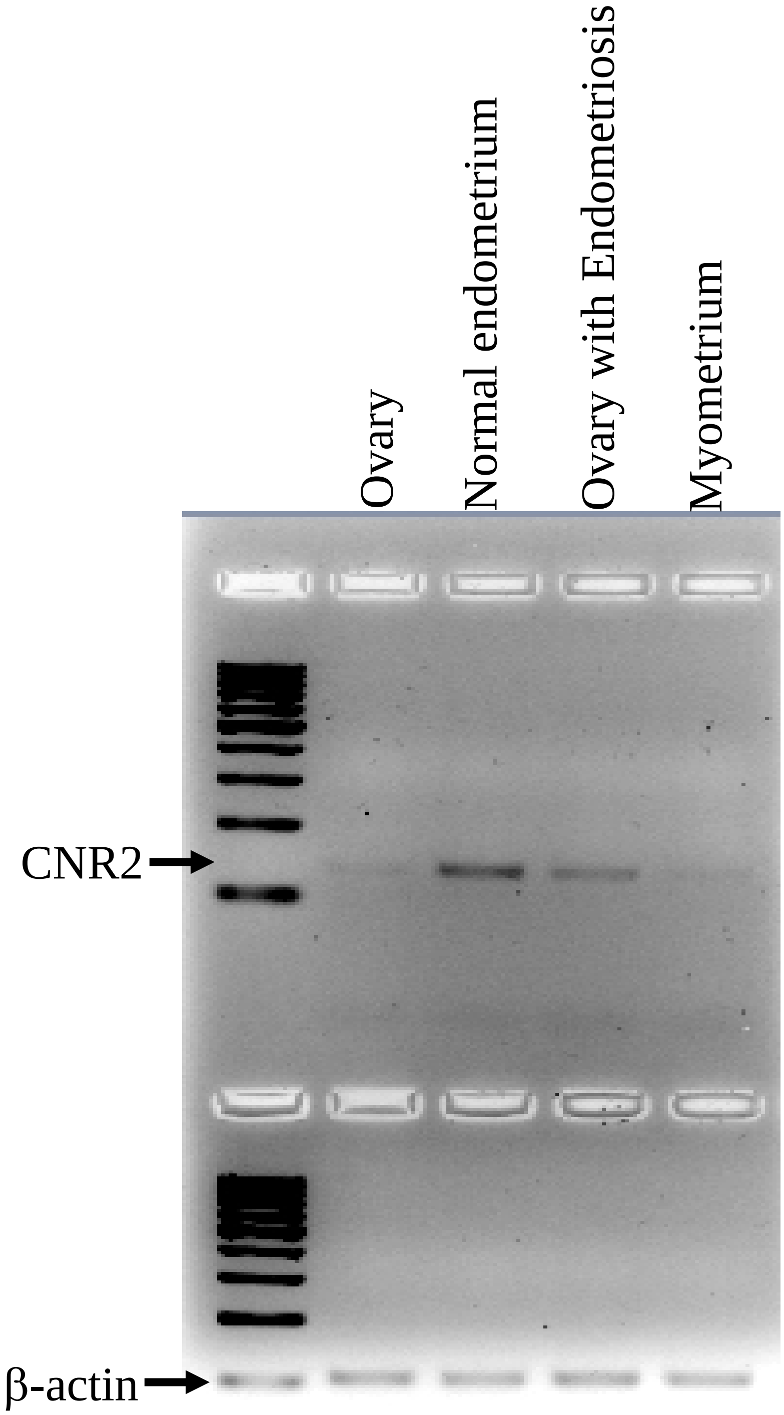

Supplementary figure 5
